# Murine leukemia virus (MLV) replication monitored with fluorescent proteins

**DOI:** 10.1186/1743-422X-1-14

**Published:** 2004-12-20

**Authors:** Katja Sliva, Otto Erlwein, Alexandra Bittner, Barbara S Schnierle

**Affiliations:** 1 Institute for Biomedical Research, Georg-Speyer-Haus, Paul-Ehrlich-Str. 42-44, 60596 Frankfurt/Main, Germany; 2 Paul-Ehrlich-Institute, Paul-Ehrlich-Str. 51-59, 63225 Langen, Germany

## Abstract

**Background:**

Cancer gene therapy will benefit from vectors that are able to replicate in tumor tissue and cause a bystander effect. Replication-competent murine leukemia virus (MLV) has been described to have potential as cancer therapeutics, however, MLV infection does not cause a cytopathic effect in the infected cell and viral replication can only be studied by immunostaining or measurement of reverse transcriptase activity.

**Results:**

We inserted the coding sequences for green fluorescent protein (GFP) into the proline-rich region (PRR) of the ecotropic envelope protein (Env) and were able to fluorescently label MLV. This allowed us to directly monitor viral replication and attachment to target cells by flow cytometry. We used this method to study viral replication of recombinant MLVs and split viral genomes, which were generated by replacement of the MLV *env *gene with the red fluorescent protein (RFP) and separately cloning GFP-Env into a retroviral vector. Co-transfection of both plasmids into target cells resulted in the generation of semi-replicative vectors, and the two color labeling allowed to determine the distribution of the individual genomes in the target cells and was indicative for the occurrence of recombination events.

**Conclusions:**

Fluorescently labeled MLVs are excellent tools for the study of factors that influence viral replication and can be used to optimize MLV-based replication-competent viruses or vectors for gene therapy.

## Background

Efficient and long-lasting gene delivery is the major challenge in the development of vectors for gene therapy. Replication-competent retroviruses (RCRs) encoding suicide genes linked via an internal ribosome entry site (IRES) offer a significant advantage over replication-deficient vectors in cancer gene therapy, since they are able to spread efficiently *in vivo *[1-4]. Uncontrolled virus spread is, however, associated with serious risk of adverse events due to viral-integration mutagenesis. Therefore, for a therapeutic application, RCRs have to be equipped with additional safety features, e.g. transcription controllable by exogenous agents or viral entry restricted to the diseased cells. The selective delivery of a therapeutic gene by targeting retroviral entry would immensely reduce unfavorable side effects and ease the clinical application of gene therapy. The ecotropic MLV envelope protein does not recognizes receptors on human cells. An obvious challenge has been to extend the host range of vectors carrying the ecotropic envelope glycoprotein to a predetermined human cell type. This change in host range requires the inclusion of a novel attachment site and the induction of fusion via a novel receptor interaction. It has been shown before that it is possible to modify ecotropic Env and change its binding specificity, however, the efficient triggering of the membrane fusion or the escape from endosomes of viral particles targeted to e.g. epidermal growth factor (EGF)-receptor is still missing [5,6]. The further development of such targeted vectors requires the understanding of the mechanisms that are involved in adsorption and internalization of retroviruses.

Investigating murine leukemia virus (MLV) replication is technically inconvenient because MLV infection does not cause a cytopathic effect in the infected cell. Viral replication can only be studied by immunostaining, measurement of reverse transcriptase activity or syncytia formation. We have developed a tool to simplify these analyses. We generated an MLV tagged with a fluorescent envelope protein, which allows viral replication and Env attachment to target cells to be followed by flow cytometry. This method will be useful for optimizing RCRs or retroviral vectors for gene therapy.

## Results

### Construction of GFP-tagged MLVs and their replication

**Figure 1:**
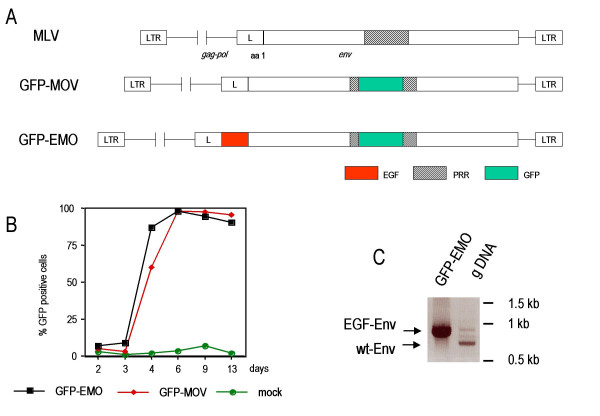
Generation and replication of the GFP-Env-tagged viruses. (A) Schematic representation of the GFP-Env-tagged viruses. EGF, epidermal growth factor; PRR, proline rich region; GFP, green fluorescent protein; L, signal peptide.(B) Viral replication kinetic in transfected NIH3T3 cells monitored by the percentage of GFP-positive cells.(C) PCR analysis of genomic DNA from FLY-Jet cells transfected with GFP-EMO. The N-terminal sequences of the EGF-Env gene were analyzed by PCR using the primers MLV-5'-Env and BS-5. GFP-EMO plasmid DNA was used as a positive control and gave rise to a 900 bp fragment. Predominantly faster migrating fragments were amplified from genomic DNA (gDNA) of GFP-EMO transfected FLY-Jet cells 32 days after transfection.

We previously constructed a modified ecotropic murine leukemia virus (Mo-MLV) bearing the green fluorescent protein (GFP) from *Aequoria victoria *in its envelope. A replication competent ecotropic MLV variant was generated (GFP-EMO) that had the 53 aas of the epidermal growth factor (EGF) fused to the N-terminus of Env and the GFP sequences inserted into the proline-rich region (PRR) [7]. We deleted the EGF sequences by replacing a Pfl MI fragment of GFP-EMO with wt sequences. This resulted in a replication-competent virus expressing the chimeric GFP-Env protein (GFP-MOV) (Fig. 1A). NIH3T3 cells were transfected with 10 μg plasmid DNA encoding GFP-MOV or GFP-EMO using the calcium-phosphate procedure and were cultured for 13 days. Viral replication was monitored as GFP-positive cells by flow cytometry. As indicated in Figure 1B, both viruses replicate with similar kinetics. Untransfected NIH3T3 cells did not show green fluorescence.

Sequestering of EGF-Env-containing viral particles has been described before [8,9]. Viral particles containing EGF-Env were rapidly trafficked to endosomes and became degraded. This effect was dominant over the normal entry pathway, because mouse cells expressing the ecotropic receptor and the EGF-receptor showed a severely decreased infectivity of EGF-Env containing vectors [8]. We were interested, if replication competent GFP-EMO might be useful to select viral variants able to escape the degradation in the endosomes. Transfection of GFP-EMO into cells expressing only the EGF-receptor (A431, COS-7) did not result in viral replication (data not shown). Therefore, GFP-EMO and GFP-MOV were transfected into FLY-Jet cells [10], which express the human EGF-receptor and the receptor for ecotropic MLV. Viral replication of GFP-EMO could be observed in FLY-Jet cells, although strongly delayed, after 10 days only 7.4 % of the cells were GFP-positive. After 38 days, all cells were GFP-positive and the N-terminus of the Env gene was analyzed by PCR amplification of genomic DNA isolated from infected cells. Predominantly a band migrating faster than the GFP-EMO fragment was amplified (Figure 1C), which was verified by sequence analysis to contain wt Env sequences. The less abundant, slower migrating fragments still contained the EGF sequences in Env. This confirms the sequestering of EGF-Env containing retroviral particles via the EGF-receptor. The selection of viruses able to escape the endosomal degradation was not possible and shows that degradation of viral particles in the endosomes favors the selection of wt Env-containing MLV, which escapes the sequestering by EGF-receptor.

### Cell binding of GFP-tagged MLV

**Figure 2:**
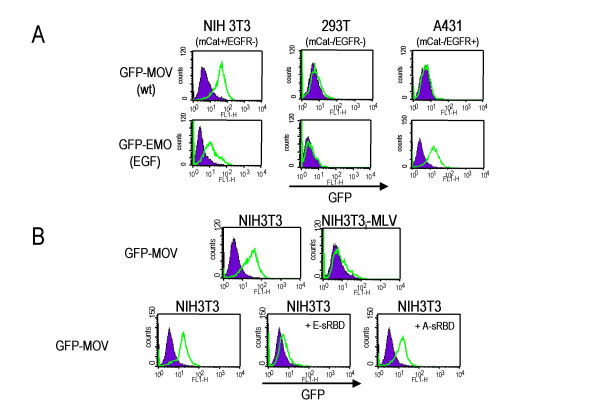
Binding of GFP-Env to cells. (A) Supernatants of GFP-EMO- or GFP-MOV-infected NIH3T3 cells were incubated with the indicated target cells and analyzed by flow cytometry. Binding of GFP-Env was detected by a shift to green fluorescence (FL-1).(B) Supernatants from GFP-MOV-infected NIH3T3 cells were incubated with the indicated target cells and analyzed by flow cytometry. Soluble receptor binding domains of the ecotropic or the amphotropic MLV Env (E-sRBD, A-sRBD) were added prior to the virus, as supernatants from 293T cells transfected with the expression constructs. After 5 mins., supernatants of GFP-MOV-infected NIH3T3 cells were added for an additional 5 mins. Binding of GFP-Env was detected by a shift to green fluorescence (FL-1). NIH3T3_i_-MLV: chronically MLV-infected NIH3T3 cells.

Viral entry is initiated by the binding of the envelope protein (Env) to the retrovirus receptor at the target cell surface. To test whether labeling of Env with GFP allows viral attachment to be monitored, we incubated supernatants of NIH3T3 cells producing GFP-EMO or GFP-MOV with cells that either express mCAT, the receptor for ecotropic MLV [11] (NIH3T3), do not express it (293T, A431) or do express the human EGF receptor (A431). As illustrated in Figure 2A, NIH3T3 cells incubated with cell culture supernatants showed a shift to green fluorescence, indicating specific binding of GFP-tagged Env to mCAT. The shift to green fluorescence could not be increased by larger amounts of viral supernatants or longer incubation times (data not shown), which shows that already after 5 min. all receptors are occupied by Env. For GFP-MOV supernatants a shift in fluorescence was only observed with mCAT-expressing cells, while GFP-EMO supernatants also produced a shift with A431 cells. This indicates additional specific binding to the EGF receptor. The shift was more pronounced on A431 cells than COS-7 cells, correlating with the amount of EGF receptor expressed by the target cells (data not shown).

The specificity of cell staining by supernatants containing GFP-MOV was further examined using chronically Mo-MLV-infected NIH3T3 cells (NIH3T3_i_-MLV). These cells have only negligible numbers of mCAT molecules on the cell surface, because Env expression leads to their retention within the cell (receptor interference). As expected, NIH3T3_i_-MLV cells produced no shift when incubated with GFP-MOV supernatants (Fig. 2B). Furthermore, binding of GFP-MOV supernatants could be inhibited by preincubation of NIH3T3 target cells with a soluble Env fragment containing the receptor binding domain (sRBD) derived from the ecotropic Env [12], but not with the equivalent sRBD derived from the amphotropic Env [12], which binds to a different receptor (Fig. 2B). This shows that GFP-tagging can be used to investigate Env-binding properties by flow cytometry.

### Replication of semi-replicative retroviral vectors

The size of a retroviral genome is limited to roughly 11 kb. The capacity for the insertion of a therapeutic gene for gene therapy is, however, increased by the use of semi-replicative retroviral vectors (SRRVs), where the *gag*/*pol *and *env *genes are split between two viral genomes. We constructed split viral genomes and used fluorescent proteins to monitor the replication of the resulting SRRVs.

**Figure 3:**
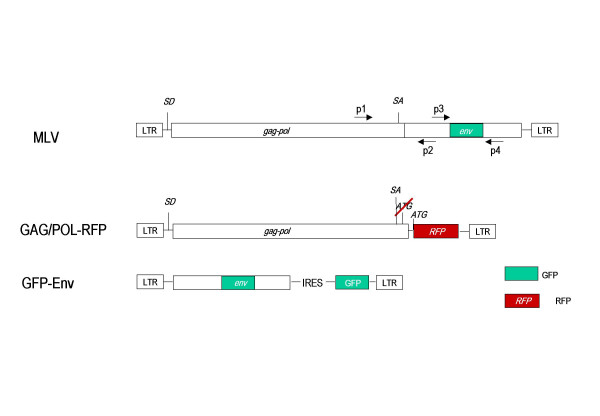
Schematic representation of fluorescently labeled semi-replicative retroviral vectors. The *env *open reading frame was replaced with the gene for red fluorescent protein (RFP) in the gag/pol-expressing construct, GAG/POL-RFP, and GFP-tagged Env was expressed from a packagable vector (GFP-Env). Positions of primers used to analyze the appearance of replication-competent viruses and the stability of the inserted GFP sequences by polymerase chain reactions (PCR) are indicated as p1 to p4. SA: splice acceptor site; SD: splice donor site.

**Figure 4:**
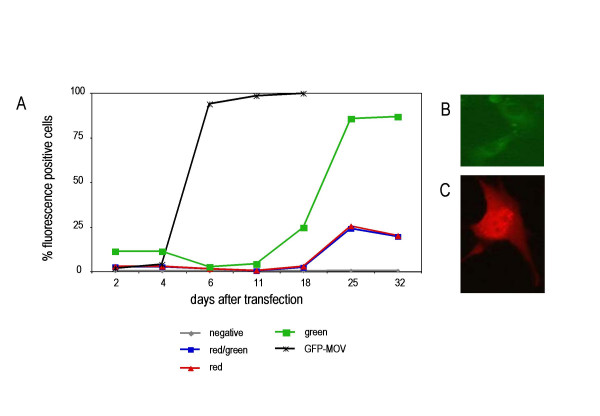
eplication of semi-replicative retroviral vectors. (A) Replication of semi-replicative retroviral vectors in transfected NIH3T3 cells, monitored by detection of green, red or double fluorescent cells by flow cytometry.(B) NIH3T3 cells expressing GFP-Env. The green fluorescence of the GFP-Env fusion protein can be detected in regions surrounding the nucleus (ER/golgi) and in the plasma membrane.(C) NIH3T3 cells expressing GAG/POL-RFP. RFP expression can be detected all over the cell, since RFP is not fused to a viral protein and is able to freely diffuse.

**Figure 5:**
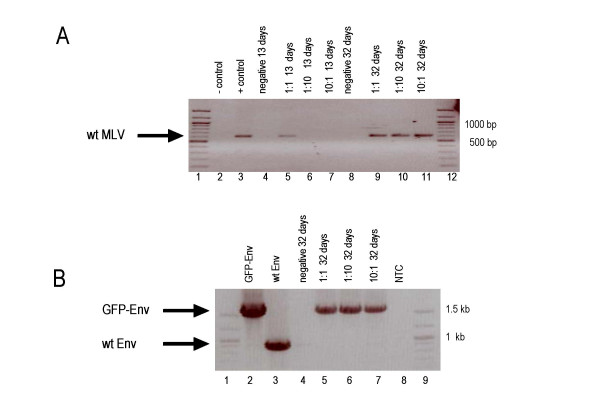
PCR analysis of genomic DNA from NIH3T3 cells transfected with semi-replicative retroviral vectors. (A) The generation of full-length MLV genomes was analyzed by PCR using the primers p1 and p2 (see Fig. 4). Full-length MLV generates an 800 bp PCR fragment, semi-replicative retroviral vectors should not give rise to a DNA fragment because the primers do not bind to the same genome. DNA was transfected in different molar ratios as indicated. The first number indicates the molar ratio of the gag/pol plasmid and the second the Env encoding plasmid.(B) The stability of the GFP sequences inserted into the Env gene was analyzed by PCR using the primers p3 and p4 (see Fig. 4). The gfp-env sequence gives rise to a 1.5 kb fragment and wt env to an 800 bp fragment. Untransfected NIH3T3 cells were cultured in parallel and analyzed identically. The data are given as negative at days 13 and 32. NTC, no template control.

A packagable MLV Gag/Pol expression vector, GAG/POL-RFP, was generated by deleting of the *env *gene and replacing it with the red fluorescent protein (RFP) (Fig. 3). RFP is encoded by the spliced mRNA and its expression can be monitored by red fluorescence (Fig. 4C). The GFP-Env protein was cloned into the retroviral vector pczCFG5 IEGZ (Lindemann, unpublished) (Fig. 3). This vector has additional GFP sequences linked via an IRES element, but GFP expression derived from IRES-GFP in transduced cells is barely detectable. GFP expressing cells always showed staining of the endoplasmatic reticulum (ER)/Golgi and plasma membrane but not of the nucleus. This is the expected pattern for Env, indicating that the green fluorescence detected derived from GFP-Env (Fig. 4B). Co-transfection of equal amounts of both plasmids into NIH3T3 cells resulted in the spread of both genomes, which was detecteable by the appearance of green and red fluorescence (Fig. 4A, green, red and double positive). Separation of the viral genomes strongly delayed viral growth and we did not observe 100% double-positive cells in any of the transfections. Since the expression of Env in the target cell leads to receptor down-regulation (receptor interference), Env-expressing cells should no longer be transducible. This could explain the selected appearance of GFP-positive cells, but their rapid increase starting day 12 also points towards the generation of full-length MLV genomes containing GFP-Env. We therefore, analyzed the integrity of the viral genomes by PCR. Both split genomes were co-transfected in different ratios into NIH3T3 cells and genomic DNA was isolated at the time points indicated in Figure 5. Primers derived from the *pol *and the *env *regions (p1, p2; Fig. 3) were used to study the generation of full-length MLV from the split genomes. As indicated in Figure 5A, lane 3, a 600 bp fragment can be amplified from full-length MLV DNA using these primers. The split genomes do not give rise to a DNA fragment, because the primer binding sites are on separate genomes (Fig. 5A, lane 2). After 13 days of culture, the appearance of a full-length MLV recombinant could be observed when the vector genomes were co-transfected in a ratio of 1:1 (gag/pol:env) (Fig. 5A, lane 5) and after 32 days, wt MLV could be detected in all samples (Fig. 5A, lanes 9, 10 and 11). This illustrates that full-length MLV was generated from the split viral genomes after prolonged passage.

In addition, we examined the stability of the GFP-tagged Env in the split genome approach. As shown in Figure 5B, PCR analysis with primers flanking the GFP sequences in Env (p3, p4; Fig. 3) clearly demonstrated that GFP-Env is stable and the GFP sequences were not deleted from the viral genome after 32 days of culture (Fig. 5B, lanes 5, 6 and 7).

## Discussion

Our data demonstrate that labeling the MLV Env with a fluorescent protein is an easy method of monitoring MLV replication and the attachment of Env to target cells. This is especially useful for the development of novel cancer gene therapies that use replication-competent MLV encoding a cytotoxic gene [3]. Labeling Env with GFP in the PRR leaves the 3' untranslated region at the Env boundary available for the insertion of IRES-linked therapeutic genes [1]. These recombinant viruses could be monitored by GFP expression and would allow the study of replication kinetics *in vitro *and *in vivo*. The biodistribution of replication-competent viruses in animal models and their safety for cancer treatment could, thereby, be assessed.

A further improvement of replication-competent viruses would be tumor cell-specific entry. The inclusion of tumor-specific ligands into Env is one option to potentially expand the ecotropic host range of MLV to human tumor cells [6,5]. Ecotropic MLV containing GFP-tagged Env can be used to analyze the receptor-dependent binding of the viral Env proteins to target cells. Labeling Env in the PRR leaves the N-terminus or the receptor binding site [13] available for further insertions of ligands to target tumor cell specific receptors. The use of GFP-tagged Env to determine receptor binding is very simple and in addition GFP-tagged Envs are helpful for the identification of recombinant viruses from retroviral library screens. GFP-Env fusions will therefore be very useful for the development of targeted vectors and as a screening system for retroviral-receptor antagonists. However, selecting EGF-Env containing MLV on cells that express both receptors (EGF- and ecotropic receptor) did not permit the isolation of a virus with an EGF-receptor specific tropism. EGF sequences were deleted from the viral genome in this setting. EGF sequences in Env, however, did not alter the replication kinetics in mouse fibroblasts (Fig. 1), which further indicates that targeting retroviruses to membrane spanning receptor tyrosine kinases inactivates retroviral particles.

In our experiments using semi-replicative retroviral vectors, we found that a rapid increase in GFP-positive cells correlated with the appearance of recombinations and the formation of full-length MLV genomes. This indicates that semi-replicative vectors have to be improved to avoid intergenomic recombination before they can be considered to be used for gene therapy. The recombinants did contain the GFP-Env gene, providing further proof that insertion of GFP into the proline-rich region of Env did not interfere with viral fitness.

## Conclusions

Fluorescently labeled MLVs are excellent tools for the study of factors that influence viral replication and can be used to optimize MLV-based vectors or viruses for gene therapy. This method is not limited to ecotropic Env, but can be extended to amphotropic MLV, since it has been shown recently that the amphotropic MLV Env can also be tagged with GFP [14].

## Methods

### Cell lines

NIH3T3, A431, 293T and COS-7 cells were grown in Dulbecco's modified Eagle's medium (Gibco) supplemented with 10% fetal calf serum, 4 mM L-glutamine, 100 U/ml penicillin and 100 μg/ml streptomycin at 37°C in 10% CO_2_.

### Plasmids

The construction of GFP-EMO has been described previously [7]. GFP-MOV was generated by replacing a Pfl MI fragment of pGFP-EMO with wt MLV sequences using standard cloning procedures [15]. GAG/POL-RFP was generated starting with the genomic MLV clone, pKAΔenv-egfp, which contains a 30 nucleotide-linker with an Sfi I-site introduced at position 5893 (all positions according to GenBank Accession No. J02255) and an additional Sfi I-site at position 5389 removed by mutation. The start codon of MLV *env *(position 5777) was deleted to allow translation to start at the inserted GFP sequence [16]. We replaced GFP with RFP, which was introduced as a Sfi I-Cla I fragment. GFP and RFP sequences were derived from vectors purchased from Clontech (BD Biosciences Clontech, Heidelberg, Germany)

### Transfections

Plasmids encoding the MLV genomes or soluble receptor binding fragments (sRBDs) [12] were transfected using the calcium phosphate procedure [15]. For the sRBDs, supernatant was collected two days after transfection, filtered through a 0.45 μm pore filter (Millipore, Eschborn, Germany) and 1 ml was used per binding assay.

### Cell binding assay

Supernatants of tissue culture cells were collected, filtered through a 0.45 μm pore filter (Millipore, Eschborn, Germany) and added to target cells. After 5 min. at room temperature, the cells were spun down, redispersed in PBS and immediately monitored by fluorescence-activated cell sorting (FACScan, Becton Dickinson, Heidelberg) using the Cellquest software.

### Fluorescence-activated cell sorter (FACS) analysis

Green fluorescence protein (GFP) expression was monitored by a shift to green fluorescence (FL-1) and red fluorescent protein (RFP) by a shift to red (FL-2). FACS analysis was performed with FACScan (Becton Dickinson, Heidelberg) using the Cellquest software.

### Polymerase chain reaction (PCR)

Genomic DNA was isolated after proteinase K digestion and phenol/chloroform extraction. PCR was performed using the manufacturers protocol (Qiagen, Hilden, Germany).

N-terminal EGF-Env sequences were analyzed using the primers BS-5: 5'-TCT GAG TCG GAT CCC AAA TGT AAG and MLV-5'-Env: 5'-TAA CCC GCG AGG CCC CCT AAT CC, which amplified a 899 bp fragment from GFP-EMO and a 726 bp fragment from wt MLV. The generation of full-length genomes was analyzed using the primers p1: 5'-GAA TAG AAC CAT CAA GGA GAC and p2: 5'-CTC GAG AAG CTT AGT ACT GA, which amplify a 600 bp fragment from full-length MLV. No fragment should be amplified from the semi-replicative vectors, because the primers bind to genes on separate constructs. The stability of the GFP-Env fusion gene was analyzed using the primers p3: 5'-GTC AGT AAG CTT CTC GA and p4: 5'-GGT TTT GTC AGG ACT GGT GAG, which amplify a 1.5 kb fragment from gfp-env and an 800 bp fragment form wt env.

## Competing interest

The author(s) declare that they have no competing interests.

## Authors' Contributions

Katja Sliva and Alexandra Bittner performed the experiments. Katja Sliva, Otto Erlwein and Barbara Schnierle participated in the design of experiments, oversight of the conduction of the experiments, and in the interpretation of the results.
